# *Luteibacter flocculans* sp. nov., Isolated from a Eutrophic Pond and Isolation and Characterization of *Luteibacter* Phage vB_LflM-Pluto

**DOI:** 10.3390/microorganisms11020307

**Published:** 2023-01-24

**Authors:** Ines Friedrich, Alisa Kuritsyn, Robert Hertel, Rolf Daniel

**Affiliations:** 1 Genomic and Applied Microbiology and Göttingen Genomics Laboratory, Institute of Microbiology and Genetics, Georg-August-University of Göttingen, 37077 Göttingen, Germany; 2 FG Synthetic Microbiology, Institute of Biotechnology, BTU Cottbus-Senftenberg, 01968 Senftenberg, Germany

**Keywords:** *Luteibacter*, phage host system, phage isolate, *Rhodanobacteraceae*

## Abstract

*Luteibacter* is a genus of the *Rhodanobacteraceae* family. The present study describes a novel species within the genus *Luteibacter* (EIF3^T^). The strain was analyzed genomically, morphologically and physiologically. Average nucleotide identity analysis revealed that it is a new species of *Luteibacter*. In silico analysis indicated two putative prophages (one incomplete, one intact). EIF3^T^ cells form an elliptical morphotype with an average length of 2.0 µm and width of 0.7 µm and multiple flagella at one end. The bacterial strain is an aerobic Gram-negative with optimal growth at 30 °C. EIF3^T^ is resistant towards erythromycin, tetracycline and vancomycin. We propose the name *Luteibacter flocculans* sp. nov. with EIF3^T^ (=DSM 112537^T^ = LMG 32416^T^) as type strain. Further, we describe the first known *Luteibacter*-associated bacteriophage called vB_LflM-Pluto.

## 1. Introduction

The genus *Luteibacter* is part of the family *Rhodanobacteraceae,* which belongs to the γ-subclass of the Proteobacteria. The family contains 17 genera, *Aerosticca*, *Ahniella*, *Aquimonas*, *Chiayiivirga*, *Denitratimonas*, *Dokdonella*, *Dyella*, *Frateuria*, *Fulvimonas*, *Luteibacter*, *Oleiagrimonas*, *Pinirhizobacter*, *Pseudofulvimonas*, *Rehaibacterium*, *Rhodanobacter*, *Rudaea* and *Tahibacter,* of which two are not validly published (*Denitratimonas* and *Pinirhizobacter*) [1]. The genus *Luteibacter* was established by Johansen et al. [2] based on the species *Luteibacter rhizovicinus* DSM 16549^T^. It currently comprises five species of which three are validly published: *L. rhizovicinus* DSM 16549^T^ [2], *L. yeojuensis* DSM 17673^T^ [3,4], *L. anthropi* CCUG 25036^T^ [4], as well as *L. jiangsuensis* [5] and *L. pinisoli* [6]. Members of the *Luteibacter* genus were isolated from various environments such as rhizospheric soil [2,6], greenhouse soil [3] and human blood [4]. They are described as motile, aerobic Gram-negatives with a rod-like shape and yellow coloring. Further, they are catalase- and oxidase-positive and urease-negative.

To date, *Luteibacter*- or even *Rhodanobacterceae*-associated phages are unknown. Phages or bacteriophages are viruses that infect bacteria. While temperate phages can incorporate into the bacterial genome, lytic phages begin multiplying directly after infection. The temperate phages replicate their incorporated genome alongside the host genome, leading to a prophage and a lysogenic bacterium. Through the addition of its genetic material, a prophage can provide new abilities, defending the host from infection by related and unrelated viruses [7].

In a previous study, we were able to isolate an environmental *Luteibacter* sp. nov. strain from a eutrophic pond located in Göttingen, Germany. The *Luteibacter* strain was isolated as a prospective model strain to investigate the local viral diversity associated with it. Despite the fact that 16S rRNA gene analysis validated its species assignment, no additional characterization was performed [8].

Here, we describe a novel environmental *Luteibacter* isolate, which was characterized morphologically, physiologically and genomically. In addition, we investigated the potential of the host strain to access the environmental diversity of *Luteibacter*-associated phages.

## 2. Materials and Methods

### 2.1. Luteibacter flocculans EIF3 Strain Isolation, DNA Extraction and 16S rRNA Gene Sequencing

*Luteibacter flocculans* EIF3^T^ (Figure S1) was isolated from the surface water of a eutrophic pond located at the North Campus of the Georg-August University in Göttingen, Germany (51°33′29″ N 9°56′41″ E 173 m, collected on 24 September 2018) [8]. This study was conducted at a public pond in Göttingen that required no specific permissions; 25 mL LB (1% peptone from casein, 0.5% yeast extract, 0.5% NaCl) was used as a culture medium. DNA was extracted and the 16S rRNA gene sequenced was as described by Friedrich et al., 2021 [8].

### 2.2. Sequencing, Assembly and Annotation of Bacterial and Phage Genome

Friedrich et al. 2021 describe the genome sequencing, assembly and annotation procedures. Briefly, Illumina paired-end sequencing libraries were generated using the Nextera XT DNA Sample Preparation kit. For sequencing, the MiSeq System and Reagent Kit version 3 (2 × 300 bp) were used according to the manufacturer’s instructions (Illumina, San Diego, CA, USA) [8]. For Nanopore sequencing, the Ligation Sequencing Kit (SQK-LSK109) and the Native Barcode Expansion Kit EXP-NBD114 (Oxford Nanopore Technologies, Oxford, UK) were utilized [8]. The same kit was used to prepare total and specific nucleic acids from the bacteriophage. To remove proteins, 5 µL of Proteinase K (20 mg/mL) were added to 300 µL of 2X T and C Lysis. This solution was applied to 300 µL of phage suspension. The pure viral genomic DNA was extracted from total nucleic acids using RNase A (DNase free).

CRISPRCasFinder [9] was used to identify potential CRISPR areas. Assembled bacterial and viral genomes as well PHASTER-predicted prophages were quality-checked with CheckM v1.1.2 [10] and CheckV v1.0.1, respectively [11]. Genome annotation was performed using the Prokaryotic Genome Annotation Pipeline v4.13 (PGAP) [12].

Raw bacteriophage reads were quality-processed using Trimmomatic v0.39 [13] and paired-end reads were merged using FLASH v1.2.11 [14]. The quality-processed reads served as input for the Unicycler v0.4.9 assembly pipeline in normal mode [15], which consisted of Spades v3.13.0 [16], makeblastdb v2.11.0+ and tblastn v2.11.0+ [17], bowtie2-build v2.4.4 and bowtie2 v2.4.4 [18], SAMtools v1.12 [19], java v.11.0.13 [20] and Pilon v1.23 [21]. The quality of assembly was evaluated using QualiMap v2.2.2 [22]. Annotations were performed using VIBRANT [23] and InterProScan v5.55-88.0 [24].

The whole-genome sequence of *Luteibacter flocculans* EIF3^T^ has been submitted to GenBank under the accession number CP063231. The BioSample (SAMN16456042) is part of the BioProject with the accession number PRJNA669578. The raw reads have been submitted to the NCBI SRA database with the accession numbers SRR12951264 (Oxford Nanopore) and SRR12951265 (Illumina), as well as BioProject PRJNA669578. The strain was deposited at the DSMZ (Deutsche Sammlung von Mikroorganismen und Zellkulturen, Braunschweig, Germany) with the collection number DSM 112537 and at the BCCM/LMG (Belgian Coordinated Collections of Microorganisms) with the collection number LMG 32416. The whole-genome sequence of *Luteibacter*-associated bacteriophage vB_LflM-Pluto is available at GenBank under the accession number ON529861 at GenBank.

### 2.3. Luteibacter flocculans *sp. nov.* EIF3^T^ Phylogenetic Classification 

The Genome Taxonomy Database Toolkit (GTDB-Tk) v1.0.2 [25] as well as whole-genome-based phylogeny with the Type (Strain) Genome Server (TYGS [26], accessed on 10 July 2022) were used to provide an initial taxonomic classification of the *Luteibacter flocculans* isolate. The ANIm method, which is provided by pyani v0.2.10 [27], was used with a species boundary of 95% ANI for in-depth phylogenetic analysis [25]. Based on the DSMZ and the NCBI, the genome of the isolate was compared to all available type strain and reference genomes (accessed on 10 July 2022) comprising *Frateuria flava* (GCF_017837635), *F. defendens* (GCF_001182895), *Dyella solisilvae* (GCA_003351225), *D. kyungheensis* (GCF_016905005), *Luteibacter pinisoli* (GCF_006385595), *L. jiangsuensis* (GCA_011742555), *L. yeojuensis* (GCA_011742875), *L. anthropi* (GCA_011759365), *D. terrae* (GCA_004322705), *Fulvimonas soli* (GCA_003148905), *D. thiooxydans* (GCA_001641285), as well as *L. rhizovicinus* (GCA_001010405).

### 2.4. Genomic Characterization

BlastKOALA v2.2 [28] was used to study the metabolic capacities of *Luteibacter flocculans* (Figure S2). AntiSMASH v6.0.0 [29] was employed to identify putative secondary metabolite biosynthetic gene clusters. PHASTER [30] was utilized to identify putative phage regions. Resfams v1.2.2 [31] was applied to examine presence of antibiotic resistance genes.

### 2.5. Cell Morphology and Gram Staining Techniques

Microscopy (Primo Star, Zeiss, Carl Zeiss Microscopy, Jena, Germany) was used to examine the morphology of single colonies after 72 hours of growth on LB solid medium (Fluka, Munich, Germany). Hucker’s crystal violet, an iodine and safranin solution and 1-propanol were used for Gram staining [32]. Microscopic images and stains were processed and analyzed using the software ZEISS Labscope (Carl Zeiss).

### 2.6. Bacterial and Phage Isolate Transmission Electron Microscopy

The morphology of *Luteibacter flocculans* and *Luteibacter* phage vB_LflM-Pluto was studied using transmission electron microscopy (TEM). The digital Micrograph software (Gatan GmbH, Munich, Germany) was used for imaging. *Luteibacter flocculans* was cultivated overnight in liquid LB medium at 30 °C. A negative staining was then conducted using a 5 µL cell or phage suspension. The suspension was mixed with an equal amount of diluted 0.5% (for bacterial isolate) or 1% (for viral isolate) phospho-tungstic acid (3% stock, pH 7). The mixture was transferred to a vaporized carbon mica for one minute. Before placing the mica on a thin copper-coated grid (PLANO GmbH, Marburg, Germany), it was gently cleaned with demineralized water. The coated grids were allowed to dry at room temperature and examined with a Jeol 1011 TEM (Georgia Electron Microscopy, Freising, Germany).

### 2.7. Determination of Salt Tolerance and Optimal Temperature

EIF3^T^ was incubated at 30 °C in 4 mL LB medium adjusted with 0 and 5 g/L NaCl and 10 to 60 g/L NaCl in increments of 10 g to determine its salt tolerance. The optical density of the cell suspensions was measured at 600 nm (OD_600_) using the Ultraspec 3300 pro photometer (Amersham Pharmacia Biotec Europe GmbH, Munich, Germany). At the start of the experiment, the OD_600_ of the cell suspensions was set to 0.1 [33], followed by a 12-h incubation period at 30 °C and 180 rpm in an Infors HT shaker (Orbitron, Einsbach, Germany). To determine growth, the OD_600_ was measured after 12 h of incubation [33]. Every measurement was carried out in biological replicates. The temperature optimum was determined by culturing the isolate in 4 mL LB-0 medium under shaking (180 rpm) at 10, 20, 30, 37, 40 and 50 °C. The cultures’ starting OD_600_ was set to 0.1. After 12 h, the optical cell density of EIF3^T^ was determined. R studio version 4.0.0 [34] and the ggplot2 package v3.3.6 [35] were used to visualize the data.

### 2.8. Growth Kinetics Determination

The cell growth quantifier (CGQuant 8.1; Aquila Biolabs GmbH, Baesweiler, Germany) was used to evaluate growth kinetics in liquid cultures under shaking (180 rpm) for 47 h at 30 °C. 250 mL shake flasks were filled with 25 mL of EIF3^T^ culture in LB-0 medium (final OD_600_ of 0.1) and placed for measurement on the CGQuant sensor plate. Experiments were carried out using three biological replicates. The CGQuant uses a dynamic method of backscattered light measurement, allowing real-time monitoring of growth in liquid culture [36]. All data were plotted with R studio version 4.0.0 [34] and the ggplot2 package v3.3.6 [35].

### 2.9. Antibiotic Resistances and Metabolic Activity

For assessment of metabolic activity, API ZYM and API 20 NE tests (BioMérieux, Nuertingen, Germany) were used. Both tests were carried out according to the manufacturer’s instructions. Catalase activity was measured with 3% H_2_O_2_ [37]. Antibiotic resistances with discs and strips (Oxoid, Thermo Fisher Scientific, Waltham, MA, USA) were determined using a soft-agar (0.4% (*w*/*v*) agarose in LB medium) overlay technique. Discs and strips contained ampicillin (25 µg), kanamycin (30 µg), oxytetracycline (30 µg), rifampicin (2 µg), streptomycin (10 µg), vancomycin (30 µg), tetracycline (0.015–256 µg) and erythromycin (0.015–256 µg). 2.5 mL of soft agar with a final OD_600_ of 0.1 was utilized. Discs or strips containing an antibiotic substance were then added on top of the soft agar. Antibiotic resistances were determined after overnight incubation at 30 °C.

### 2.10. Examination of Plaques 

The approach described by Willms and Hertel, 2016 [38] and Willms et al., 2017 [38,39] was used for phage enrichment. Sewage samples were collected in February 2022. In order to identify plaque morphologies such as clear or turbid, plaque size and halo presence, plaque assays generally require the ability of the host to grow in bacterial lawns [40]. Phages were isolated via agar overlay plaque assay as described elsewhere [40] using host-specific culture media for the base agar (1.5% (*w*/*v*) agar) and overlay (0.4% (*w*/*v*) agarose). Infected overlay plates were incubated overnight at 30 °C. Individual phages appeared as morphologically distinct plaques, which were picked with a sterile toothpick and transferred into 500 µL sterile LB-0 medium. Reinfection was repeated three times to purify the phage strain. 

### 2.11. Naming of Bacteriophage Isolate

The bacteriophage was named according to Adriaenssens and Brister’s informal guide [41]. As a result, vB stands for virus of bacteria, Lfl for the host organism *L. flocculans*, M for the virus family *Myo*-morphotype and Pluto is an individual name. As a result, the complete name of the virus is vB LflM-Pluto, abbreviated to Pluto in the following.

## 3. Results and Discussion

### 3.1. Morphological Characterization

**Figure 1:**
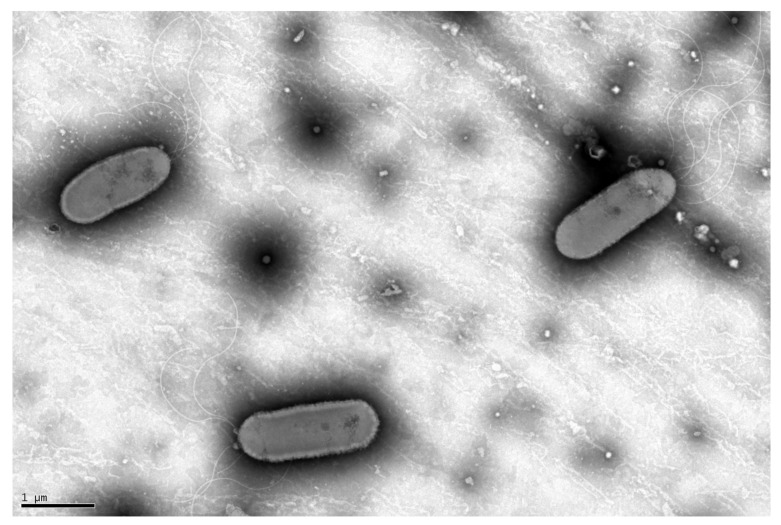
Transmission electron micrograph of EIF3^T^. The micrograph depicts the rod-shaped, flagellated morphotype of the *Luteibacter* EIF3 isolate. The image was taken using TEM after 24 h of cell growth at 30 °C in LB medium followed by negative staining.

EIF3^T^ colonies were spherical and yellow with an average diameter of 1.93 mm on solid LB medium. The ability of *Luteibacter flocculans* sp. nov. to flocculate was apparent during growth in liquid LB or TSB media (Figure S1). Gram staining of EIF3^T^ resulted in red/pink cells indicating a Gram-negative bacterial species. Cells were straight rods with rounded ends and ranged from 5.3 to 5.8 µm in size (Figure 1). The isolate matched typical morphological features of the family *Rhodanobacteraceae* such as motility via polar flagella, a cell size ranging from 1 to 4.5 µm and rod-shaped cells with rounded ends [42].

### 3.2. Physiological Characterization

**Figure 2:**
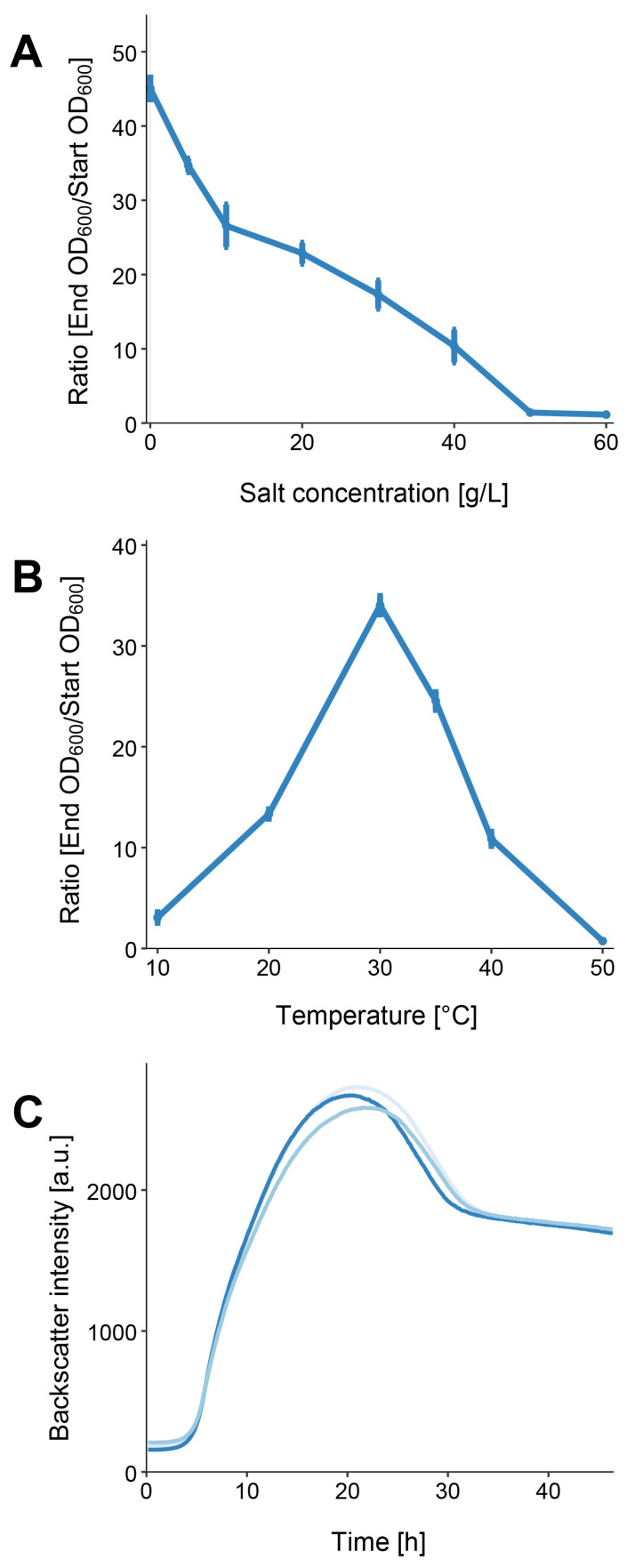
*L. flocculans* sp. nov. growth characteristics. (**A**) Growth of EIF3^T^ in 4 mL LB medium after 12 h of incubation at 180 rpm and 30 °C with different salt concentrations. (**B**) EIF3^T^ growth at various temperatures in LB-0 medium after 12 h of incubation at 180 rpm. (**C**) Growth characterization of EIF3^T^ in 25 mL LB-0 medium at the optimal temperature (30 °C). Triplicate measurements were conducted. Standard deviations in (**A**,**B**) are indicated by the error bars. In (**C**) various shades of blue represent each biological replicate.

EIF3^T^ grew in LB medium with up to 4% (*w*/*v*) NaCl, with optimal growth achieved in the absence of added NaCl (Figure 2A). The strain is a mesophilic organism since it can grow at temperatures between 20 and 40 °C. The largest cell densities were observed at 30 °C with an OD_600_ of 3.403 (which is a ratio of 34.033, (Figure 2B). This observation is consistent with results derived from closely related strains [2].

We determined the bacterial growth of EIF3^T^ at the optimal temperature and salt concentration. The lag phase lasted for approximately 3.5 h. It was followed by a 10 h log-phase and a transient phase with diminished growth. After approximately 21 h of incubation, the highest cell densities were recorded. The doubling time of our isolate was 221 min and the growth rate µ was 0.19 h^−1^.

Using the API ZYM and the API 20 NE assays, the metabolic capabilities of EIF^T^ were examined. Twenty distinct enzyme activities were identified for the novel *Luteibacter* isolate using API ZYM. In six cases, no enzyme activity was detected. Activities of alkaline phosphatase, esterase, esterase lipase, leucine arylamidase, valine arylamidase, cysteine arylamidase, acid phosphatase, naphthol-AS-BI-phosphohydrolase, β-galactosidase, α-glucosidase, β-glucosidase and N-acetyl-β-glucosaminidase were recorded. Corresponding genes were identified in the genome (Table S1). In addition, *Luteibacter flocculans* shared features with closely related bacteria such as *L. rhizovicinus*, *L. yeojuensis* and *L. jiangsuensis* [2,3,5].

EIF3^T^ was oxidase- and catalase-positive, which is characteristic of certain *Rhodanobacteraceae* family members [42]. Table 1 provides an overview of the enzymatic activities of the strain and closely related bacteria from TYGS [26]. According to the antibiogram, EIF3^T^ was resistant to erythromycin (up to 4 µg/disc), tetracycline (up to 1 µg/disc) and vancomycin (30 µg/disc). Resfams in silico analysis [31] identified genes encoding an ABC transporter for erythromycin or vancomycin (PRJNAA669578|IM816_002307) and a tetracycline inactivation enzyme (IM816_003460; Table S2). The in silico analysis of EIF3^T^ confirmed the measured antibiotic resistances (Table 1).

Further, it suggested, that EIF3^T^ can generate secondary metabolites such as aryl-polyene xanthomonadin (Table S3). Xanthomonadin is a yellow membrane-bound pigment, which is insoluble in water. Rajagopal et al. discovered that xanthomonadin may protect *Xanthomonas oryzae* against photodamage [45]. Moreover, this discovery is consistent with the characteristics of the family *Rhodanobacteraceae* [42].

Fatty acid analysis confirmed typical *Luteibacter* characteristics of our isolate and related strains. The most abundant fatty acids were branched fatty acids iso-C_15:0_ with 18.3%, iso-C_17:1_ ω9c with 29.4% and iso-C_17:0_ with 18.2% (Table 2). These corresponded to the main branched fatty acids of the *Luteibacter* described by Johansen et al. [2].

### 3.3. Genome Characterization

The closed genome of EIF3^T^ comprised one circular chromosome (4,299,254 bp) with a GC content of 64.82%. It encoded 3672 putative proteins, 59 rRNAs and 49 tRNAs. No CRISPR regions and plasmids were identified (Table 3).

**Figure 3:**
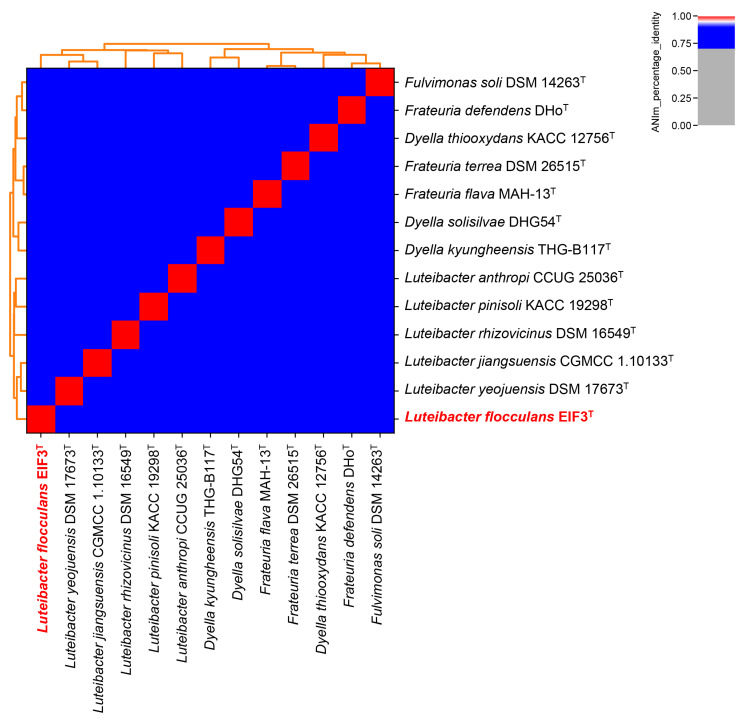
Genome-based classification of *Luteibacter flocculans* EIF3^T^. All genome sequences from available type strains (T) listed in the TYGS database [26] were examined. Pyani [27,47] was used to calculate relatedness employing the ANIm technique and default settings. EIF3^T^ is highlighted in bold red letters.

GTDB-Tk was used for the genome-based taxonomic classification of strain EIF3^T^ (Data File S1) [25]. It demonstrated an average nucleotide identity (ANI) of approximately 96% to the most closely related species, *Luteibacter* sp. UNCMF366Tsu5.1 (ANI value of 96.48). The digital DNA-DNA hybridization value (dDDH) calculated by the Type Strain Genome Server (TYGS) is 39.9% in comparison to *L. jiangsuensis*. As the new species criterion for dDDH is less than 70% [46], this suggested that our strain is a potential new species (Figure S2; Table S4). Figure 3 shows an ANI-analysis of 12 most closely related type strain genome sequences in the TYGS database [26] (data in Table S5). No clustering with any other described *Luteibacter* strain was recorded. EIF3^T^ shares 85.52% ANI with *L. yeojuensis* DSM 17673^T^, 85.41% with *L. jiangsuensis* CGMCC 1.10133^T^, 84.73% with *L. anthropic* CCUG 25036^T^ and 84.60% with *L. rhizovicinus* DSM 16549^T^.

*Luteibacter flocculans* sp. nov. EIF3^T^ was therefore considered as a novel type strain within the *Luteibacter* genus.

### 3.4. Prophages Analysis

EIF3^T^ represented a prospective host system for the investigation of the environmental diversity of phages. Thus, the capabilities to host prophages was of particular interest in this study. Initial analysis of prophage regions with PHASTER [30] revealed two putative prophage regions (region 1, 1,300,438–1,322,193; region 2, 1,306,588–1,352,491). The regions were 21.7 and 45.9 kb in size. It was estimated that region 1 was incomplete (20.97% completeness estimation by CheckV [11]) and region 2 intact (62.83% completeness estimation by CheckV [11]). In addition, region 1 was part of region 2 (Table S6). Since prophages can provide phage-resistance to the host, the genome of a potential host strain for phage isolation must have a low number of prophages or none at all [7], which is applicable for EIF3^T^. A repressor protein cI was predicted (locus tag: IM816_005715) in both prophages. Bacterial cells which harbor a lysogenic lambda phage are immune to further lambda phage infections [48]. In addition, a gene encoding RecA-dependent nuclease (locus tag: IM816_005740), which is also encoded by the temperate bacteriophage P1 [49]. was predicted. The terminase (large subunit) gene (locus tag: IM816_005805) involved in the initiation of phage DNA packaging into the prohead indicated that prophage 2 is an active prophage. The phage portal protein (locus tag: IM816_005810) binding to the terminase subunit to form the packaging machinery was also identified [50]. A phage major capsid protein (locus tag: IM816_005820) indicated an icosahedral head structure, placing the prophage withing the *Claudoviricetes*. The tail assembly chaperone protein (locus tag: IM816_005855) suggested a *Sipho*-morphotype. Lastly, a gene coding for a tail tape protein (locus tag: IM816_005865) was also present. During infection, the protein determines tail length and facilitates DNA transit [51].

### 3.5. Phage Isolation and Characterization

*Luteibacter flocculans* has proven to be an organism with minimal requirements for cultivation and proliferation. This provided a solid foundation for its prospective use in molecular biology. To evaluate its viability as a host strain for environmental phage isolations. EIF3^T^ was infected with a viral suspension obtained from raw sewage. To determine cell infection, an overlay plaque test was performed. To avoid redundancies during phage isolation, picked plaques were assessed by identifying the unique genetic restriction patterns of each phage isolate. This approach led to the isolation of a novel *Luteibacter*-associated phage.

**Figure 4:**
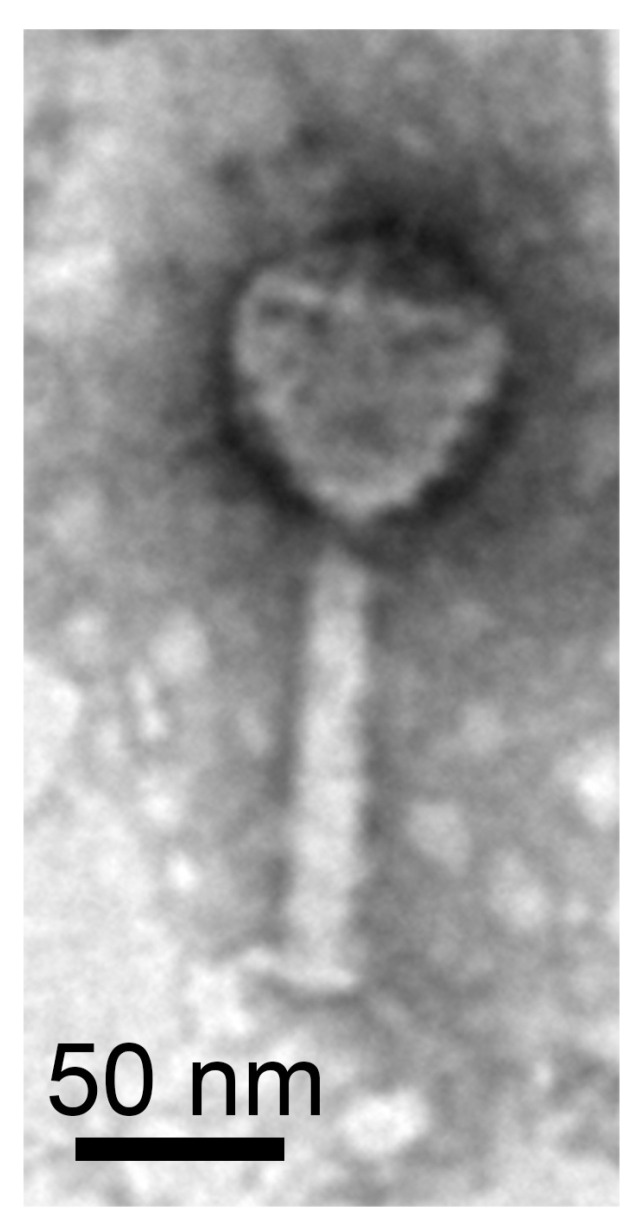
*Luteibacter*-associated bacteriophage vB_LflM-Pluto.

Transmission electron microscopy shows a *Myo*-morphotype head-tail morphology (Figure 4). The phage consisted of an icosahedral head, contractile tail and tail spikes. The diameter of the capsid was 75 nm and the length of the tail 110 nm, resulting in a total length of 185 nm. We sequenced the viral DNA and assembled the genome with a high coverage of 690.1-fold. The assembled viral genome exhibited a size of 67,528 bp, a completeness estimate of 100% (according to the CheckV report [11]) and a G + C content of 57.7% (host G + C content 64.8%). A total of 99 CDS of which 79 encode hypothetical proteins were detected. We detected genes similar to phage-related genes. These included a spike protein, showing the highest sequence identity to the corresponding protein of the *Xanthomonas* phage FoX6, which also has a *Myo*-morphotype [52]. Further, the terminase (large subunit), baseplate protein J, major capsid protein and DNA polymerase shared an average sequence identity of 60% to the corresponding proteins of FoX6. The host *Xanthomonas* (family: *Lysobacteraceae*) belongs as *Luteibacter* to the order Lysobacterales. The terminase (large subunit) suggested an icosahedral head morphotype while the baseplate protein J indicated a *Myo*-morphotype. An overview of all genes is shown in Table S7. Resulting from the morphological and genomic investigations, our phage was named vB_LflM-Pluto (vB = virus of bacter, Lfl = *L. flocculans*, M = *Myo*-morphotype, Pluto = specific phage name). Our results represented the first description of a phage from the bacterial *Rhodanobacteraceae* family. In addition, we showed that *Luteibacter flocculans* sp. nov. is a suitable host strain for phage isolation.

While *Luteibacter* phage LflM-Pluto is of the *Myo*-morphotype, the prophage of *Luteibacter flocculans* likely has a *Sipho*-morphotype. Both showed no gene or protein similarities and therefore were not genetically related. No genes encoding for antibiotic resistances were detected in either phage genome.

## 4. Conclusions

The results demonstrated the suitability of the novel *Luteibacter* species *L. flocculans* for the isolation of environmental phages. The isolation and characterization of a novel *Luteibacter*-associated phage vB_LflM-Pluto, the first documented *Luteibacter*-associated phage, further supported this.

### Description of Luteibacter flocculans *sp. nov.*

*Luteibacter flocculans* (floc.cuĭlans N.L. part. adj. *flocculans*, flocculating, pertaining to the organism’s ability to flocculate in liquid cultures). *L. flocculans* cells were Gram-negative rod-shaped, 2.0 µm long and 0.7 µm wide. They did not form spores and were motile by means of lophotrichous bacteria. After 72 h of growth on LB medium, colonies were 1.93 mm in diameter and showed yellow pigmentation. Cells grew at 10–45 °C (optimum 30 °C) and at 0–4% NaCl (optimum without addition of NaCl). The strain was catalase and oxidase positive. Cell growth occurred on R2A agar, TSA and LB agar. The strain was susceptible to erythromycin, tetracycline and vancomycin, but not to ampicillin, kanamycin, oxytetracycline, rifampicin and streptomycin. It utilized esculin/ferric citrate, D-glucose (assimilation), D-mannose, N-acetyl-D-glucosamine and malic acid employing the API 20NE test system. Alkaline phosphatase, esterase, esterase lipase, lipase, leucine arylamidase, valine arylamidase, cysteine arylamidase, acid phosphatase, naphthol-AS-BI-phosphohydrolase, β-galactosidase, α-glucosidase, β-glucosidase and N-acetyl-β-glucosaminidase activities were detected with API ZYM test system. In Table 1 and Table 2, additional phenotypic characteristics are depicted.

The type strain EIF3^T^ (=DSM 112537^T^ = LMG 32416^T^), was isolated from a eutrophic pond located on the North Campus of the Georg-August University in Göttingen, Germany. The major fatty acids were C_15:0 iso_, C_17:0 iso_ and summed feature 9 (C_17:1 iso_ ω9c). The genome of the type strain showed a DNA G + C content of 64.8 mol%.

## Figures and Tables

**Table 1:** Phenotypic differences between strains EIF3^T^ and phylogenetically related species *L. yeojuensis* DSM 17673^T^, *L. jiangsuensis* CGMCC 1.10133^T^, *L. antrophi* CCUG 25036^T^ and *L. rhizovicinus* DSM 16549^T^. Taxa: 1, strain *L. flocculans* EIF3^T^; 2, strain *L. yeojuensis* DSM 17673^T^ [3], 3, strain *L. jiangsuensis* CGMCC 1.10133^T^ (data from [5,43]); 4, strain *L. antrophi* CCUG 25036^T^ (data from [4] and BacDive [44] accessed on 31 July 2022); 5, strain *L. rhizovicinus* DSM 16549^T^ (data from [2] and BacDive [44] accessed on 31 July 2022); +, Positive; –, negative; n/a, data not available.

Characteristics	*L. flocculans* EIF3^T^	*L. yeojuensis* DSM 17673^T^	*L. jiangsuensis* CGMCC 1.10133^T^	*L. anthropi* CCUG 25036^T^	*L. rhizovicinus* DSM 16549^T^
**Source of isolation**	Eutrophic pond	Rhizosphere soil	Soil	Human blood	Rhizosphere soil
**Motility**	+	+	–	+	+
**Temperature (°C)**					
Range	10–45	5–37	4–42	15–37	5–30
Optimum	30	28	25–30	28	17.5
**NaCl (g/L)**					
Range	0–40	0–50	0–40	n/a	0–30
Optimum	0	n/a	n/a	n/a	15
**Enzymatic activity**					
Alkaline phosphatase	+	+	+	n/a	+
Esterase	+	+	n/a	n/a	–
Esterase lipase	+	+	n/a	+	–
Lipase	+	–	+	n/a	–
Leucine arylamidase	+	+	n/a	n/a	+
Valine arylamidase	+	+	n/a	n/a	+
Cysteine arylamidase	+	+	n/a	n/a	–
Trypsin	–	–	n/a	n/a	–
α-Chymotrypsin	–	–	n/a	n/a	–
Acid phosphatase	+	+	n/a		+
Naphthol-AS-BI-phosphohydrolase	+	+	+	n/a	+
α-Galactosidase	–	+	n/a	n/a	+
β-Galactosidase	+	+	+	+	+
β-Glucuronidase	–	–	n/a	n/a	–
α-Glucosidase	+	+	+	n/a	+
β-Glucosidase	+	+	n/a	n/a	+
N-Acetyl-β-glucosaminidase	+	+	+	n/a	–
α-Mannosidase	–	–	n/a	n/a	–
α-Fucosidase	–	–	n/a	n/a	–
**Utilization of**					
Potassium nitrate	–	–	+	n/a	–
L-Tryptophane	–	–	n/a	n/a	–
D-Glucose (fermentation)	–	–	n/a	–	–
L-Arginine	–	–	+	n/a	–
Urea	–	–	–	n/a	–
Esculin/ferric citrate	+	+	+	n/a	+
Gelatin	–	+	+	–	+
4-Nitrophenyl-β-D-galacto-pyranoside	–	–	n/a	n/a	–
D-Glucose (assimilation)	+	+	–	+	+
L-Arabinose	–	–	+	n/a	–
D-Mannose	+	+	+	+	+
D-Mannitol	–	–	–	+	–
N-Acetyl-D-glucosamine	+	+	n/a	+	+
D-Maltose	–	+	+	n/a	–
Potassium gluconate	–	–	n/a	+	–
Capric acid	–	–	n/a	n/a	–
Adipic acid	–	–	n/a	n/a	–
Malic acid	+	–	–	+	–
Trisodium citrate	–	–	n/a	n/a	–
Phenylacetic acid	–	–	n/a	n/a	–
**Oxidase**	+	+	+	+	+
**Catalase**	+	+	+	–	+
**Resistance to**					
Ampicillin	–	n/a	n/a	n/a	n/a
Erythromycin	+	n/a	n/a	n/a	n/a
Kanamycin	–	n/a	n/a	n/a	n/a
Oxytetracycline	–	n/a	n/a	n/a	n/a
Rifampicin	–	n/a	n/a	n/a	n/a
Tetracycline	+	n/a	n/a	n/a	n/a
Streptomycin	–	n/a	n/a	n/a	n/a
Vancomycin	+	n/a	n/a	n/a	n/a
**G + C %**	64.8	63.0	63.6	65.3	63.0

In bold: Sorted by categories.

**Table 2:** Composition of cellular fatty acids (%) in strain EIF3^T^ and phylogenetically related species *L. yeojuensis* DSM 17673^T^, *L. jiangsuensis* CGMCC 1.10133^T^, *L. antrophi* CCUG 25036^T^ and *L. rhizovicinus* DSM 16549^T^. Taxa: 1, strain *L. flocculans* EIF3^T^; 2, strain *L. yeojuensis* DSM 17673^T^ [3,4], 3, strain *L. jiangsuensis* CGMCC 1.10133^T^ (data from [5]); 4, strain *L. antrophi* CCUG 25036^T^ (data from [4]); 5, strain *L. rhizovicinus* DSM 16549^T^ (data from [4]); –, not detected/not reported.

Fatty acid	*L. flocculans* EIF3^T^	*L. yeojuensis* DSM 17673^T^	*L. jiangsuensis* CGMCC 1.10133^T^	*L. anthropi* CCUG 25036^T^	*L. rhizovicinus* DSM 16549^T^
Unknown 11.799	–	2.3	–	0.8	2.2
iso-C11:0	4.3	3.8	4.7	3.6	4.0
iso-C11:0 3-OH	4.1	4.2	1.6	2.9	3.9
iso-C13:0	0.2	–	–	0.4	0.5
iso-C12:0 3-OH	0.1	1.0	–	–	–
iso-C14:0	0.2	1.1	–	–	–
C14:0	0.1	–	–	0.5	0.4
iso-C13:0 3-OH	3.2	2.4	2.6	1.2	2.7
iso-C15:0	18.3	14.5	24.0	21.7	17.0
anteiso-C15:0	8.0	6.9	9.7	2.4	4.0
iso-C16:0	2.8	21.3	2.2	0.5	0.8
Summed feature 3 *	5.8	5.2	4.1	6.5	9.2
C16:0	2.1	1.8	4.2	5.6	6.5
iso-C17:1 ω9c	29.4	26.5	20.3	23.8	24.4
iso-C17:0	18.2	14.9	20.2	27.0	22.4
anteiso-C17:0	1.3	1.6	1.2	0.9	0.6
C18:0	0.1	–	0.8	0.5	–
iso-C17:0 3-OH	0.7	0.8	–	–	0.5

* Summed feature 3 contains C_16:1_ ω7c and/or iso-C_15:0_ 2-OH.

**Table 3:** Genome statistics of the *Luteibacter flocculans* sp. nov. EIF3^T^ chromosome.

Features	*Luteibacter flocculans* sp. nov. EIF3^T^
Genome size (bp)	4,299,254
GC content (%)	64.82
Coverage	280.1-fold
Coding sequence (CDS)	3672
rRNA genes	59
tRNA genes	49
ncRNA	4
CRISPR	0
Prophage(s)	2
Completeness estimate *	100%

* According to the CheckM [10] report.

## Data Availability

The whole-genome shotgun project of *Luteibacter flocculans* sp. nov. EIF3^T^ has been deposited at GenBank under the accession numbers CP063231 and the BioProject accession number PRJNA669578. BioSample accession number is SAMN16456042. The raw reads have been submitted to the NCBI SRA database with the accession numbers SRR12951264 (Oxford Nanopore) and SRR12951265 (Illumina), as well as BioProject PRJNA669578. The genome of *Luteibacter* phage vB_LflM-Pluto has been deposited at GenBank under the accession number ON529861.

## Supplementary Materials

The following supporting information can be downloaded at: https://www.mdpi.com/article/10.3390/microorganisms11020307/s1, Figure S1: Flocculation of EIF3^T^ in LB medium; Figure S2: Phylogenetic classification of *Luteibacter flocculans* EIF3^T^. Table S1: KEGG Mapper reconstruction result of *Luteibacter flocculans* EIF3^T^ chromosome [26,53,54]; Table S2: Resfams prediction of *Luteibacter flocculans* EIF3^T^; Table S3: List of putative biosynthetic gene clusters in *Luteibacter flocculans* EIF3^T^; Table S4: List of putative biosynthetic gene clusters in *Luteibacter flocculans* EIF3^T^ [26]; Table S5: Phylogenetic analysis for *Luteibacter flocculans* EIF3^T^; Table S6: PHASTER analysis of *Luteibacter flocculans* EIF3^T^; Table S7: Genes and putative their functions of *Luteibacter*-associated phage vB_LflM-Pluto and its closest relatives; Data File S1: GTDB-Tk of *Luteibacter flocculans* sp. nov. isolate.
