# Supplementary material for: Luteibacter flocculans sp. nov., Isolated from a Eutrophic Pond and Isolation and Characterization of Luteibacter Phage vB_LflM-Pluto
Source: Microorganisms. 2023 Jan 24;11(2):307. doi: 10.3390/microorganisms11020307 (PMC9965599; doi:10.3390/microorganisms11020307)
Supplement: Supplementary file 1 [file microorganisms-11-00307-s001.zip › Figures_S1-S2.pdf]

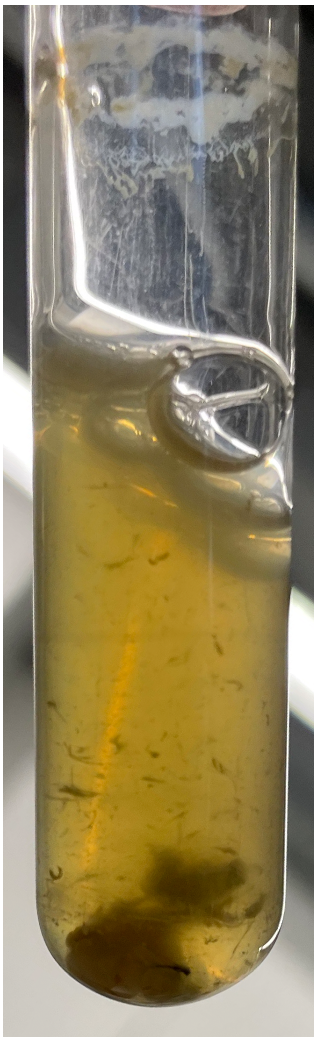

Figure S1. Flocculation of $EIF3^T$ in LB medium.

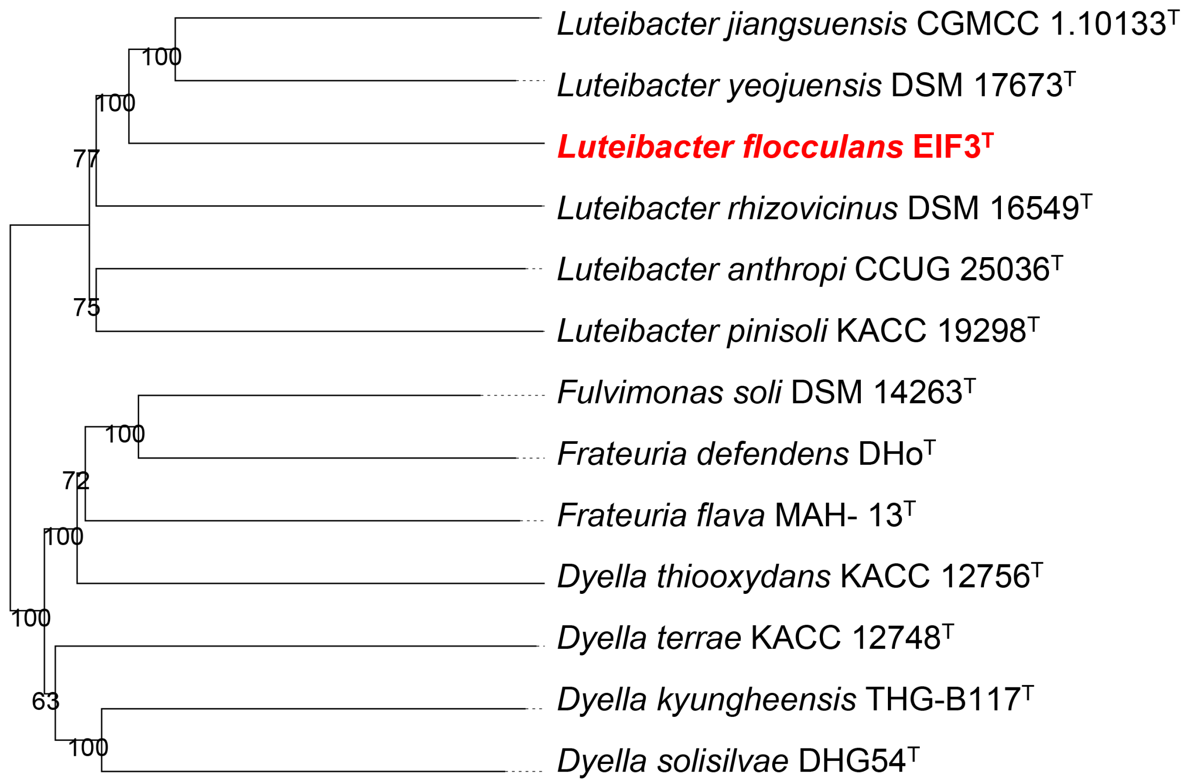

Figure S2. Phylogenetic classification of *Luteibacter flocculans* EIF3^T. The 12 closest related type strain genomes were used for phylogenetic analysis as described by TYGS (Meier-Kolthoff and Göker, 2019). The tree was inferred with FASTME 2.1.6.1 (Lefort et al., 2015) using Genome Blast Distance Phylogeny (GBDP) distances calculated from genome sequences. The branch lengths are scaled in terms of GBDP distance formula d_5. The numbers above the branches are GBDP pseudo-bootstrap support values of >60% from 100 replications, with an average branch support of 85.1%. The tree was rooted at the midpoint (Farris, 1972).

Meier-Kolthoff JP, Göker M. TYGS is an automated high-throughput platform for state-of-the-art genome-based taxonomy. Nat Commun. 2019;10(1):2182.

Lefort V, Desper R, Gascuel O. FastME 2.0: a comprehensive, accurate, and fast distance-based phylogeny inference program. Mol Biol Evol. 2015;32(10):2798–800.

Farris JS. Estimating phylogenetic trees from distance matrices. Am Nat. 1972;106(951):645–68.
